# The Age of Attraction: Age, Gender and the History of Modern Male Homosexuality

**DOI:** 10.1111/1468-0424.12437

**Published:** 2019-07-12

**Authors:** Kate Fisher, Jana Funke

## Introduction

In 1902, founder of the Berlin Institute for Sexual Science, Magnus Hirschfeld, developed a ‘psychobiological questionnaire’ to investigate human sexuality.^1^ The section labelled ‘sexual instinct’ [*Geschlechtstrieb*] asked: ‘What approximately are the highest and lowest limits in age of the people to whom you feel drawn, or is age of no importance to you?’^2^ Hirschfeld's interest in age as a driver of sexual attraction reflects broader sexual scientific attempts to map an erotics of age.^3^ This article explores how and why the relative age of sexual subjects was theorised by British and German sexual scientists in the late nineteenth and early twentieth centuries. It demonstrates that questions around age preoccupied sexual science and argues, more specifically, that constructions of male homosexuality were driven by anxieties about interactions between children or adolescents and older men.^4^ In so doing, the article reveals the interrelationship between sexological debates about homosexuality and scientific explorations of childhood sexuality and adolescent development. It shows that age needs to be acknowledged as a crucial category shaping sexual scientific debate and structuring understandings of modern homosexuality.^5^

As is well known, the erotic charge provided by age difference was central to many culturally prominent eighteenth‐ and nineteenth‐century understandings of same‐sex desire.^6^ European and American elites interpreted classical (predominately Ancient Greek) cultures as providing affirmative models of youthful male beauty and intimate attachment between older and younger men or boys.^7^ While precise differences in age were rarely specified, partners were separated on the basis of maturity into active (*the erastes*) and passive roles (*the eromenos*). Moreover, attraction to younger same‐sex partners was not seen to exclude opposite‐sex relationships: men who desired male youths could also be married to women. The influence of this material on individual identities can be found, for example, in sexological case studies or letters received by prominent Hellenic writers.^8^

At the same time, this idealisation of age‐differentiated erotics reinforced existing associations between same‐sex activity and the sexual misuse of younger people by older men.^9^ In response, some sexual scientists sought to tease apart age‐structured and same‐sex relationships. These authors defined the male homosexual through his exclusive inborn attraction to other consenting adults. This article shows that this influential construction of the modern homosexual as a discrete congenital type emerged in direct response to considerations around age.^10^ It also demonstrates that, notwithstanding the emergence of this model, questions about the impact of sexual encounters with older men on a younger person's sexual development continued to be debated within sexual science, creating dialogue between theorisations of homosexuality and concurrent studies of childhood sexuality.^11^ As such, investigations of male homosexuality continued to revolve around urgent questions about how and when same‐sex desire emerged and what impact sexual experiences in youth had on later desires. These debates remained at the centre of early twentieth‐century sexual science in Britain and Germany, complicating the inborn model and securing the centrality of age as a key category in modern articulations of sexuality.

## Same‐sex desire and the seduction of youth

Throughout the nineteenth century, male same‐sex acts were frequently associated with the sexual coercion of young people in news media and forensic, legal and literary writings. The assumption that same‐sex relations were violations of youth fundamentally shaped sexual scientific investigations of same‐sex desire and informed intersecting explorations of childhood and adolescent sexual development.^12^ Later nineteenth‐century constructions of homosexuality were inextricably linked to concerns about childhood and adolescent sexuality in ways that scholars have not yet fully explored. There are two main reasons for this oversight: first, research on child sexual abuse has frequently side‐lined male‐male same‐sex behaviour, concentrating on sexual violence involving girls.^13^ Second, histories of male homosexuality have often downplayed the prominence of young people in representations of male same‐sex behaviour.^14^

From the mid‐nineteenth century, sexual science, alongside other scientific, legal and literary discussions of childhood, constructed children as at risk of physical and moral molestation.^15^ Although there was no clearly defined concept of child sex abuse before the twentieth century, minors were increasingly seen as victims of defilement rather than as sexually precocious agents.^16^ Age of consent and sexual assault laws as well as child marriage legislation in Europe and European colonies were often focused on the vulnerabilities of girls.^17^ Yet, boys were also seen as requiring protection from (often male) predators.^18^ Age of consent and sex crime legislation, introduced across Europe and North America, applied to boys as well as girls.^19^ For example, when raising the age of consent for girls from thirteen to sixteen in 1885, the British parliament added a clause protecting adolescent males. While this infamous Labouchère Amendment changed the law governing all male same‐sex acts, it was introduced to protect young males from sexual corruption.^20^

Such depictions of the violation of young males brought into focus the image of the male sexual predator. Forensic debates reinforced an association between male same‐sex desire and the abuse of youth. Of particular importance is Ambroise Tardieu's broad study of sex crimes *Étude médico‐légale sur les attentats aux moeurs* (1857), which included sections on sexual acts committed by adult men against boys under the age of twelve, and on male youth prostitution. Tardieu's analysis was not isolated.^21^ Other forensic investigations discussed similar cases and should be read as indicative of the concern for young male victims of sexual assault and not as accounts of proto‐homosexual relationships.^22^ Ludwig Julius Caspar Mende, in his *Ausführliches Handbuch der gerichtlichen Medizin* (1826), for instance, explicitly characterised unchaste acts between males as corrupting attacks on youth. In favouring the terms ‘*Knabenschande’/ ‘Knabenschändung*’ [boy defilement] and ‘*Päderastie*’, he typified same‐sex acts as age‐differentiated, involving an ‘old man weak in body and mind’ who ‘follows boys and young men with lustful eyes’.^23^

Similarly, in philosophical and anthropological writings, same‐sex sex acts were frequently depicted as exploitations of boys and young males, often in the form of prostitution. Reading these texts as depicting proto‐homosexual bonds between adults obscures how male same‐sex desire was framed as a form of youth corruption. For instance, in the 1859 edition of *Die Welt als Wille und Vorstellung*, Arthur Schopenhauer described pederasty as a ‘vice [*Laster*] of old men’ who targeted ‘male adolescents’ [*Jünglinge*].^24^ While Schopenhauer saw pederastic longings as natural and widespread among those too old or too young to produce healthy offspring, he insisted that only particularly ‘weak and brainless’ older men would act on these desires.^25^ In Richard Burton's analysis of ‘pederasty’ in his 1886 *Terminal Essay*, the ‘vice against nature’ [*le vice contre nature*] was predominantly perpetrated across the so‐called ‘sodatic zone’ on young male victims.^26^ The *Untrodden Fields of Anthropology* (1896), published under the pseudonym Jacobus X, includes similar accounts of the alleged global prevalence of ‘depraved’ ‘Arabs’, ‘Chinamen’ and ‘Europeans’ demanding ‘unnatural acts’ from ‘precocious debauchees’ whose ages (when stated) ranged from seven to twenty.^27^ As these writings suggest, the language of pederasty served, at least in part, as a locus for the articulation of anxieties about child and adolescent sexual assault rather than the exploration of adult male same‐sex desires that might be read in terms of proto‐homosexuality.^28^ Although the unstable term ‘pederasty’ could refer to male same‐sex acts, anal sex and other sexual behaviours deemed criminal, its presence in sources about child corruption reflects the prominence of anxieties about the dangers faced by young males.

Such nineteenth‐century accounts of age‐differentiated sexual exploitation resonated with sexual scientific questions about childhood and adolescent sexual development. The understanding that early sexual experiences might be physically injurious and psychologically damaging with long‐term impacts on later life was central to sexual scientific debates which constructed childhood and adolescence as crucial periods that required careful management.^29^ For Jacobus X, age‐differentiated abuse by an older man would eventually lead to ‘the depraved taste becom[ing] a pressing need’.^30^ The fear that a young person might be damaged through early sexual contact with an older same‐sex partner was reliant on wider constructions of childhood innocence as a state that could be damaged via external influences.

The intense scrutiny of childhood and adolescent sexual development intensified towards the end of the nineteenth century. Following the work of Max Dessoir, sexual scientists like Albert Moll, Havelock Ellis and Sigmund Freud began to argue that even very young children experienced sexual desires.^31^ In the 1900s, adolescence was theorised by G. Stanley Hall and others as a critical transitional period during which young people were particularly open and vulnerable to external influences.^32^ Sexual scientist Iwan Bloch suggested that the later stages of youth were ‘particularly favourable to the development of sexual aberrations and to their consolidation as habitual practices’.^33^

Nineteenth‐century investigations of childhood and adolescent sexuality and the association of male same‐sex acts with the corruption of youth had a profound impact on constructions of male homosexuality in sexual science. As the following section shows, the distinctive sexual scientific model of male homosexuality as an inborn and unchanging identity emerged in direct response to concerns about age‐differentiated bonds.

## Age difference and the construction of male homosexuality

To assuage fears about the violation of youth, homophile sexual scientists keen to present a socially acceptable model of male same‐sex relationships rejected affirmative framings of age‐differentiated relationships. They constructed an influential alternative model of homosexuality that was increasingly at odds with existing Hellenising accounts of same‐sex erotics that celebrated bonds between older and younger men.^34^

This sexual scientific model had four interrelated elements. First, male homosexuality was framed as involving adults rather than boys and children. Second, the concept of consent was marshalled to distinguish between legitimate and illegitimate same‐sex acts. Third, the homosexual was constructed as a person who was driven by gendered desires, rather than the desire for youth: the key characteristics of the homosexual became his attraction to other men and his lack of desire for women. Fourth, homosexuality was constructed as an inborn and unchanging lifelong orientation that was not caused by seduction or experiences in youth. This new model excluded men whose desire was directed towards younger partners. The category of the paedophile came into existence to describe such individuals and to establish clear distinctions between homosexuality and age‐differentiated desires.

From the mid‐nineteenth century onwards, it was increasingly difficult for homophile authors to affirm the eroticisation of age‐differentiated male‐male relations without raising the spectre of the predatory violation of vulnerable youth. By the 1890s, Oscar Wilde's court‐room defence of the ‘love that dare not speak its name’ as an admirable form of pederastic eros was doomed in the face of a prosecution that accused him of trying to seduce the nation's youth.^35^ Wilde was portrayed as a corrupter of young victims ranging from Oxford undergraduates like Bosie to teenage working‐class rent boys.^36^ The Wilde trials were one of many widely reported, nineteenth‐ and early twentieth‐century scandals that cemented associations between same‐sex desire and the abuse of youth in British and German culture.^37^

In response, many sexual scientists and reformers distanced themselves from the acceptance of age‐differentiated relationships. John Addington Symonds tried to reconcile his attraction to Hellenic models of age‐differentiated desires with his awareness that ‘Greek Love’ was often associated with youth corruption.^38^ In the early 1890s, Symonds concluded that the passion between an older and younger man could not be defended in the modern world.^39^ He adopted this position while collaborating with English sexual scientist Havelock Ellis on *Sexual Inversion* (1896), a book that argued for the decriminalisation of same‐sex relations between adult men only.^40^ Similarly, André Raffalovich developed a revised understanding of what he called ‘*unisexualité*’, which excluded people like Wilde who, he argued, were justly convicted for their criminal influence on **‘**youthful dandies’ [*jeunes vanités*].^41^

Some sexual scientists adopted new linguistic frameworks when writing about attachments between males to replace a language of pederasty that might imply the violation of younger people.^42^ Karl Heinrich Ulrichs, for example, introduced the new term ‘*Urning*’ in 1864 to move away from the language of ‘boy love [*Knabenliebe*] – [which] gives rise to the misunderstanding that the urning really loves boys, when he loves young men (*puberes*)’.^43^ While scholars have recognised Ulrichs' desire to create a non‐pejorative language, it is important to note that Ulrichs specifically sought to tease apart the Urning from the male corruptor of boys.^44^

Other sexual scientists, despite being equally concerned to distinguish homosexuality from child abuse, continued to acknowledge youth as an erotic stimulus of homosexual desire. These voices stressed that the erotic appeal of young males was analogous to the admiration that adult men frequently felt for younger women and, crucially, was not directed to the very young (a category that remained undefined). Such authors argued that men in ancient Greece entered into relationships with younger men who were sufficiently mature. German forensic scientist Johann Ludwig Casper, in his *Practisches Handbuch der gerichtlichen Medizin* (1860), called for a recognition that cases of ‘*Knaben‐ oder Jünglingsliebe*’ [the love of boys or young men] involved adult men and mature youths.^45^ Moll stressed that pederasty, even in Ancient Greece, did not involve children but adolescent men.^46^ Edward Carpenter quoted Moll approvingly in his notebooks and, ten years later, avoided using the term ‘*Knabenliebe*’ when translating Classicist Erich Bethe's *Die dorische Knabenliebe* (1907), since it suggested ‘an inferiority of age too great’.^47^ Hirschfeld agreed, explaining that ‘Paederastie’ described attraction between older and younger men who ‘can already think independently and have a beard’.^48^

Building on this argument, the concept of consent assumed a key role in attempts to legitimize same‐sex behaviours and distance homosexuality from associations with youth corruption. Hirschfeld promoted raising the age of consent laws and called for the legalisation of same‐sex sex acts provided that they were consensual and involved over‐16s.^49^ Similarly, Ellis and Symonds explained that society should tolerate expressions of ‘sexual inversion’ between ‘two male persons, who have reached years of discretion, [and who] consent together to perform some act of sexual intimacy in private’.^50^ In this way, the concept of consent was used to detach homosexuality from the corruption of youth and establish legitimate expressions of same‐sex desire.

Yet, determining the precise age at which the young person was able to consent was difficult, especially in the context of ongoing contestations around childhood and adolescent sexual development. Commenting on recent discussions of the ‘age of protection’ [*Schutzaltersfrage*], Hirschfeld remarked that consent could be variously defined on the basis of the biological age of maturity, the age of criminal responsibility, the age of citizenship or the age of military service.^51^ He proposed focusing on ‘the [individual's] ability to make decisions’ [*Entscheidungsfähigkeit*], a stance that was cited approvingly by Bloch.^52^ Such debates helped to construct the category of the consenting young person who was immune from corruption and whose involvement in sexual acts, including same‐sex acts, could be legitimated.

In constructing homosexuality as involving consenting adults, sexual scientists recast same‐sex desire as driven by the gender of the partner and not their youth. Ulrichs, for example, in the passage immediately preceding his definition of *Urnings* as distinct from men who love boys, described *Urnings* as males who ‘feel sexual attraction for men [and] sexual horror towards women’.^53^ It is easy to overlook the significance of such statements, since they appear to be straightforward articulations of same‐sex erotics. However, they were deliberate reframings of desire as fundamentally structured by a gender‐based orientation in which the age of one's partner was secondary or irrelevant: homosexuals were newly defined by their desire for other men (rather than boys) and by the absence of their attraction to women. It was a fundamental re‐articulation of male same‐sex desire as uni‐directional (the homosexual could not be attracted to both men and women) and as driven by the maleness of the object of desire (not his youth or any other trait). German naturalist Gustav Jaeger made the same point in 1878: ‘Homosexuality … rests on a quite distinctly inborn speciality of the soul … to such a degree that such persons are quite impotent before a woman’.^54^

The disassociation of male‐male same‐sex desire from the seduction of youth was reinforced by the construction of male homosexuality as inborn. According to Bloch, who came to embrace the inborn model around 1906:

Genuine homosexuality exhibits, like heterosexuality, the character of an impulse [Triebes] arising from the very nature of the personality which, from the cradle to the grave, expresses the continuity of the individual in respect also of this specific sexual tendency; there does not exist a homosexuality limited to a particular period of life, such as childhood or youth … As such, Schopenhauer's pederasty of old men [Greisenpäderastie] … and the love of Greek boys for older men do not count as [genuine] homosexuality ….^55^

Such presentations of homosexuality as inborn and unchanging allowed sexual scientists to reject the idea that it was caused by improper seduction in youth. Hirschfeld insisted that bonds between older and younger males were not damaging, since only young people who were congenitally predisposed towards homosexuality would enter into such relationships in the first place.^56^ Similarly, Symonds stressed that it was common knowledge that only inborn inverts could be permanently seduced into homosexuality, a fact tacitly acknowledged by parents who happily sent their sons to Eton and Harrow despite knowing ‘very well what goes on’.^57^

The establishment of male homosexuality as separate from the pederastic seduction of youth presented sexual scientists with a further problem: how to account for what Symonds termed ‘the depraved debauchee who abuses boys’?^58^ A solution emerged in the second half of the nineteenth century via the construction of a distinct category encompassing adults who desired children. Sexual scientist Richard von Krafft‐Ebing examined what he called ‘*pädophilia erotica*’ in an 1896 article, developing an idea originally articulated in the first edition of *Psychopathia Sexualis* (1886).^59^ Following on from Krafft‐Ebing, sexual scientists at the turn of the twentieth century increasingly began to use the term ‘paedophile’ to describe those who were pathologically predisposed to desire children, including Moll who discusses inborn ‘*Pädophile*’ in *Das Sexualleben des Kindes* (1908).^60^ The construction of this new category was at least partly driven by the desire to detach male homosexuality from child abuse. It allowed sexual scientists to draw sharp lines between the acceptable homosexual and the dangerous corruptor of youth.

To maintain this distinction, sexual scientists employed different strategies when confronted with cases of men who appeared to be homosexual and who also desired children.^61^ For instance, faced with a self‐confessed *Urning* who was accused of abusing and murdering two boys (aged five and fifteen) in 1869, Ulrichs insisted that the man was not an *Urning*, but suffered from the distinct pathological desire for ‘boys too young for sex’ [*unmannbaren Knaben*], a pathology that also afflicted heterosexual men.^62^ In 1914, Hirschfeld created the category of the paedophilic homosexual whose attraction to boys before the age of puberty was a ‘most unfortunate predisposition’ [*unglücklichste Veranlagung*] that was also exceedingly rare, affecting only 5 per cent of male homosexuals.^63^ The category of paedophilia allowed Hirschfeld and others to reject the idea that age‐differentiated desires were characteristic of all same‐sex relations and to present homosexuality as the gender‐based attraction between adult men.

Tracing the intertwined emergence of the sexological categories of the homosexual and the paedophile in the late nineteenth and early twentieth centuries sheds new light on both the history of homosexuality and the history of paedophilia. With regard to the latter, scholars have generally maintained that a distinct construction of paedophilia did not take hold until the 1970s.^64^ While it is true that the 1970s witnessed a significant increase in public debate about the paedophile, preoccupations with the age of sexual partners were nonetheless at the heart of nineteenth‐ and early twentieth‐century sexual scientific enquiry. Anxieties about age drove the construction of homosexuality as a gender‐based orientation. These concerns motivated attempts to develop a new category of paedophilia to encompass age‐structured desires and cases of child abuse, which were no longer conflated with same‐sex relations. However, as shown in the next section, the model of homosexuality as an inborn orientation in which age was irrelevant remained contested, as debates about age difference and corruption continued to shape sexual scientific investigations.

## Childhood sexuality and the causes of homosexuality

The model of male homosexuality as an inborn and unchanging gender‐based orientation did not neutralise concerns about age‐differentiated relationships and the corruption of youth. Sexual scientific writings featured abundant examples apparently indicating the inculcation of young people into homosexuality. Sexual scientists did not ignore this evidence, but drew on it in considering the causes of homosexuality and debating whether it was inborn or acquired. In so doing, they engaged with and contributed to theorisations of childhood sexuality, which further destabilised the inborn model and undermined aetiological certainty.^65^

Even sexual scientists seeking to present homosexuality as inborn acknowledged that formative experiences in youth could cause a younger person to develop same‐sex desires. Historical and anthropological materials (which had a special value for sexual scientists keen to present homosexuality as a transhistorical and transcultural form of sexual variation) drew attention to cultures that appeared to encourage same‐sex desires in the young.^66^ Ellis suggested to Symonds that in both ancient Greece and ‘Eskimo’ societies, the child was ‘brought up by its parents to sex[ual] inversion’, so that it was impossible to determine whether homosexuality resulted from an inborn predisposition or external influence.^67^ Similarly, Finnish anthropologist Edvard Westermarck, despite finding the inborn model convincing, struggled to apply it to his observations of Morocco. In *The Origin and Development of Moral Ideas* (1906), he concluded that same‐sex experiences in youth, a time when the ‘sexual instinct’ was ‘somewhat indefinite’, were capable of shaping a young person's desires in ‘a homosexual direction’.^68^ Bloch also maintained that homosexuality in young people could be the result of ‘breeding’ [*Züchtung*], giving the example of boys in male same‐sex brothels in China.^69^ He criticised reform‐oriented colleagues like German zoologist and anthropologist Friedrich Karsch for political bias in misinterpreting the anthropological evidence and holding onto an exclusively inborn model.^70^ In a letter to Bloch written in 1900, Ellis conceded that he had excluded from *Sexual Inversion ‘*vicious cases’ involving age‐differentiated same‐sex bonds that were injurious to the younger person.^71^ Such evidence continued to raise the spectre of the young person being seduced into homosexuality by an older partner.

In response, sexual scientists began to label different kinds of homosexuality. Ellis and Symonds employed the category of ‘pseudo‐homosexuality’ to understand cases in which male homosexuality was acquired.^72^ Similarly, Bloch's 1905–6 investigation of homosexuality led him to distinguish between ‘genuine’ [*echter*] inborn homosexuality and acquired pseudo‐homosexuality, a distinction and terminology that Hirschfeld adopted.^73^ Such attempts to tease apart different forms of homosexuality were meant to secure the primacy of inborn homosexuality as the only ‘true’ form of homosexuality and to exclude cases of youthful seduction. Effectively, however, these debates showed that not all homosexual desires were inborn, and kept alive the possibility that youth inculcation could cause homosexuality.

Attempts to differentiate between inborn and acquired homosexuality also drew attention to childhood as a decisive period in the individual's development. On the one hand, explorations of childhood sexuality supported the inborn model: demonstrating that homosexual tendencies were evident from the earliest stages of development eliminated seduction as a causative factor. Ellis and Symonds suggested that discerning whether an individual was a ‘true’ inborn homosexual required ‘a sufficiently minute knowledge of the subject in early life’.^74^ On the other hand, many narratives of homosexual awareness in childhood, instead of affirming sexual orientation as fixed, drew attention to the ways in which youthful sexuality was shaped by external influences, specifically older partners. In *Modern Ethics*, Symonds discusses the case of the homosexual awakening experienced by a man who was touched by ‘a comrade rather older’ than himself at the age of eight.^75^ Several case studies of male homosexuality in the first edition of *Sexual Inversion* describe fantasies or actual sexual encounters with older boys and men.^76^ As Symonds acknowledged, such cases revealed the ‘imperative impressions made on the imagination or the senses of boys during the years which precede puberty’.^77^ As such, case histories tracing sexual development from childhood onwards did not offer strong certainty regarding the inborn nature of homosexuality.

The inborn model was destabilised further by theorisations of childhood sexuality as naturally diffuse. Acknowledging that a child's desires might be undifferentiated drew attention to the developmental processes and external (potentially corrupting) forces shaping sexual orientation. Freud's accounts of sexual development, for instance, focused on the impact of young people's relationships with older persons, especially parents.^78^ For Freud, all adult sexuality was shaped by early sexual experiences; homosexuality resulted from arrested sexual development caused by developmental disturbances, including seduction by an older person.^79^

Explorations of the role of youthful seduction in causing homosexuality were not limited to psychoanalysis, but cut across different branches of sexual science.^80^ Proponents of the inborn model did not shy away from addressing the relationship between male homosexuality and childhood sexuality, despite the difficulties this posed. Hirschfeld discussed scholarship on childhood sexuality and age‐differentiated bonds in youth, including works by Dessoir, Julien Chevalier, Moll, Freud and others.^81^ He agreed that childhood sexuality was more diffuse than adult sexuality: all children, including those who became ‘strictly heterosexual’ [*scharf heterosexuell*] later on in life, experienced same‐sex desires in youth.^82^ Only the experienced sexologist, he maintained, was able to differentiate between children who fell in love with members of the same sex due to an inborn predisposition and those whose same‐sex attraction was merely symptomatic of the undifferentiated nature of childhood sexuality.^83^

Other sexologists, especially those less invested in the project of homosexual reform, concluded that it was impossible to draw a clear distinction between inborn and acquired forms of homosexuality. In response to sexologists like Chevalier or Benjamin Tarnowsky, who stressed that homosexuality could be caused by ‘moral contagion and seduction’ in youth, Moll agreed that, alongside cases of inborn homosexuality, there were instances in which young people were corrupted into permanent homosexuality due to the diffuse nature of childhood sexuality.^84^ Ultimately, he argued that theorisations of childhood sexuality demonstrated the incoherence of the inborn/acquired distinction since supposedly inborn cases, in which homosexual desires were apparent early on, might be outgrown, while cases of individuals seduced into homosexuality might nonetheless have an inborn inclination.^85^ Similarly, in the 1920s, Arthur Kronfeld, a psychiatrist and permanent staff member of Hirschfeld's Institute, concluded that the study of childhood sexuality demonstrated that ‘[t]he disjunction: inborn or acquired – is … misleading’.^86^

Even if many sexual scientists continued to foreground congenital factors in the causation of homosexuality, the inborn model was continuously challenged by arguments about childhood sexuality that drew attention to the power of external influence, including instances of youthful seduction. Despite their different investments, sexual scientists shared a fascination with the question of how and why the child developed from a state of undifferentiated sexuality into adult homo‐ or heterosexuality, which not only created divisions, but also led to ongoing dialogue between different branches of sexual science. Through these contestations, early twentieth‐century sexual science produced a range of explanatory models regarding the causes of homosexuality in which age‐differentiated relationships continued to play a significant role.

## Resisting the erasure of age‐differentiated erotics

Conflicts around the construction of male homosexuality as an age‐neutral, inborn gender orientation were amplified by those who wanted to validate age‐differentiated same‐sex relationships. Some deemed the championing of relationships between older and younger partners that had been a mainstay of homoerotic subcultures in late nineteenth‐century Germany and Britain incompatible with sexual scientific constructions of homosexuality. Others, including authors who were close to or part of sexual scientific circles, continued to champion age‐differentiated attachments, while simultaneously engaging with and participating in sexual scientific debate. Drawing on the claim that childhood sexuality was naturally undifferentiated, writers argued that same‐sex attachments in youth were not always opposed to the child's own desires or interests. They also rejected the idea that age‐structured relationships between males were necessarily corruptive. Indeed, they maintained that such relationships could be beneficial to both young people and society as a whole. These arguments resonated with early twentieth‐century sexual scientific debates. Instead of a clean break between proponents of age‐structured erotics and sexual scientists, concerns about sexual development in youth became a point of ongoing debate between these intersecting networks.

For some, the move to create an age‐neutral, inborn model of male homosexuality unnecessarily fractured a joint homophile project. British criminologist and law reformer George Ives was frustrated with a model of homosexuality that left little room for the erotics of age.^87^ In Germany, Hirschfeld's focus on the inborn model and, especially, his support for a rise in the age of consent was seen as a deliberate erasure of the ideal of age‐differentiated relationships by some allies, who had initially joined his fight against paragraph 175.^88^ These disagreements caused the 1903 split between Hirschfeld's *Scientific‐Humanitarian Committee* [*Wissenschaftlich‐Humanitäres Kommittee (WHK)*] and homophile group *Die Gemeinschaft der Eigenen* [*Society of the Unique*/*One's Own/Self‐Owners*], which was led by Adolf Brand and Benedict Friedländer.^89^ Many members of *Die Gemeinschaft* saw Hirschfeld's model of homosexuality as antithetical to their own erotic ideals, which often comprised marriage to women as well as the celebration of age‐unequal relationships between males, as expressed in their journal *Der Eigene*. The German‐British novelist and *Der Eigene* contributor John Henry Mackay, for instance, rejected sexual science altogether, criticising ‘so‐called scientific research … whose horrible confusion causes us to suffer more today than we did previously’ and castigating scientists for sacrificing the ‘love of the older man for the younger of his sex’ in pursuit of the ‘legalization of love between adults’.^90^

Yet, championing age‐structured attachments did not necessitate a rejection of sexual science, as much previous scholarship has suggested.^91^ Unlike other founders of *Die Gemeinschaft*, Friedländer did not see sexual science as incompatible with an affirmative understanding of age‐differentiated erotics. Friedländer, who studied zoology and claimed a position of scientific expertise, continued to collaborate with Hirschfeld and saw scientific methodologies, including biological and statistical approaches, as central to studies of human sexuality.^92^ He did not distance himself entirely from the inborn model.^93^ He also developed alternative scientific arguments regarding universal bisexuality and the undifferentiated nature of childhood sexuality to defend age‐differentiated relationships between men. Indeed, sexual scientific rhetoric gave Friedländer the tools to argue that attraction between two male individuals – what he called ‘physiological friendship’ – was a natural experience shared by all men.^94^ In an article published in Hirschfeld's *Jahrbuch* as well as his book *Renaissance des Eros‐Uranios* (1904), Friedländer concluded that ‘the human male is capable of experiencing the erotic drive [*Liebestrieb*] in both directions’.^95^ Friedländer mobilised scientific arguments about bisexuality and childhood sexuality (alongside other forms of evidence) to suggest that intimate same‐sex attractions were a natural part of all human life, especially during adolescence.^96^

Arguments about the undifferentiated nature of sexuality in childhood and youth made it possible to suggest that, far from being seduced, a young person might actively initiate a sexual relationship. In ‘Affection in Education’ (1899), Carpenter suggested: ‘[O]ften, indeed, I think they [age‐differentiated relationships] are begun by the younger [person] … [who] naively allows his admiration of the elder one to become visible’.^97^ This point resonated with sexual scientific arguments about childhood sexuality: Bloch acknowledged that young people could actively engage in sexual acts with older males.^98^ Although critical of Carpenter's validation of age‐differentiated attachments, Moll agreed that some sexual acts might not be contrary to the young person's own wishes and accepted that ‘precocious’ [*frühreife*] children might welcome them.^99^

Advocates for age‐structured relationships also appropriated the sexual scientific argument that young minds were susceptible to external influences, recasting such attachments as valuable. Challenging the image of older men as damaging corruptors of youth, they suggested that there was a developmental benefit provided by intimate bonds with mature partners. This argument was further supported by the theory of universal bisexuality, which suggested that same‐sex relationships were not constitutionally alien and could be enjoyed by all members of society.^100^ Writers taking this stance often accused Hirschfeld and other advocates of the inborn model of denying ‘the universal bisexual predisposition’ [*bisexuelle Veranlagung*] of all humans, falsely suggesting that same‐sex tendencies were specific to the *Urning* and presenting a damaging image of homosexual men as pathological and effeminate.^101^ According to Carpenter, all young males needed close attachments to other males since an ‘unformed mind requires an ideal of itself … to which it can cling or towards it can grow’.^102^ Rejecting Symonds’ view that age‐differentiated attachments were antithetical to modern culture, Carpenter insisted that such affectionate relationships provided a mechanism for the ‘communication of character, virtue, *arête*’.^103^ Similar ideas regarding the beneficial impact of age‐differentiated relationships were articulated by authors writing in German, including Friedländer, Elisarion von Kupffer and other contributors to *Der Eigene*, as well as Gustav Wyneken and Stefan George as part of the *Free School Community* [*Freie Schulgemeinde*] Free School Community movement.^104^

These writers stressed that age‐differentiated bonds benefited society as a whole and not just the individual. Whereas heterosexuality served the purpose of biological reproduction, male same‐sex relationships, it was claimed, furthered social, cultural and national progress.^105^ This was particularly pertinent to age‐unequal relationships that involved passing on ideals and values from an older to a receptive younger person, a figure who represented the future of society as a whole. Publications such as *Der Eigene* supported such claims through an engagement with literary and historical sources, whereas writers such as Friedländer drew on biological and zoological evidence.^106^ Accepting the idea that young people were impressionable, these writers re‐evaluated age‐differentiated bonds as a key mechanism in the progress of society itself.^107^ The powerful argument that homosexuality was not only a biological trait found among a small minority, but a relational ideal of widespread potential benefit was not ignored by reform‐oriented sexologists. Hirschfeld, for instance, valued Friedländer's views even though they existed in tension with the inborn model.^108^ Similarly, his *Jahrbuch* commended von Kupffer's and Carpenter's work for illustrating the ‘ethical and social significance of homosexuality’.^109^

Proponents of age‐differentiated bonds vigorously rejected an inborn model of homosexuality that involved the life‐long and singular attraction to adult males. While for some this stance entailed a break with sexual science, others expressed their interest in the erotics of age by engaging with and contributing to sexual science, drawing especially on the scientific frameworks of child development and universal bisexuality. These voices troubled the attempted erasure of the erotics of age from constructions of male homosexuality, and compelled even those sexual scientists most invested in the inborn model to continue to confront issues around age.

## Conclusion

Concerns about the erotics of age played a fundamental role in shaping modern understandings of male homosexuality. In particular, anxieties about age‐differentiated relationships drove the very emergence of the inborn model that presented homosexuality as impervious to external influence and corruption, and constructed gender (rather than age) as the central motivator of sexual desire. Yet, as demonstrated by questions about the individual's age preferences in Hirschfeld's questionnaire, which formed the starting point of this article, attempts to assert an age‐irrelevant gender‐based framework for theorising sexuality did not succeed in sidelining age‐differentiated forms of desire. The continued theorising of age also reflects the centrality of broader explorations of childhood and adolescent sexual development within British and German sexual science. Ongoing debates about the undifferentiated nature of childhood and adolescent sexuality and the role of external stimuli in shaping desire resulted in uncertainty regarding the causes of homosexuality. The idea that relationships between older and younger men and boys might shape sexual orientation remained live and contested. By demonstrating how age as a category of analysis modifies understandings of the history of male homosexuality, this article also makes a broader methodological intervention. It reveals the many different ways in which age, and figurations of sexual desire across the life course intersected with gender in shaping constructions of modern sexualities. As such, it calls upon historians of sexuality to incorporate age and gender alongside other crucial categories like race and class, which have not received full treatment in this article, in their future research.

## Notes

1 Elena Mancini, *Magnus Hirschfeld and the Quest for Sexual Freedom: A History of the First International Sexual Freedom Movement* (New York: Palgrave, 2010), pp. 84–5. Hirschfeld's earlier 1899 questionnaire asked whether the individual had ever felt desire for ‘immature’ [*unreife*] people. See Magnus Hirschfeld, ‘Die objektive Diagnose der Homosexualität’, *Jahrbuch für sexuelle Zwischenstufen* (1899), pp. 5–35, here p. 34. All translations are the authors’ own.

2 Magnus Hirschfeld, *Die Homosexualität des Mannes und des Weibes* (Berlin: Louis Marcus, 1914), p. 255.

3 Questions about age preference feature in other early twentieth‐century questionnaires. See Diederik F. Janssen, ‘Uranismus Complicatus: Scientific‐Humanitarian Disentanglements of Gender and Age Attractions’, *Journal of the History of Sexuality* 27 (2018), pp. 101–33, here p. 133.

4 The discussion of female same‐sex desires, which were also structured by concerns around age, lies beyond the scope of the article. We do not use terms such as ‘child’, ‘youth’ or ‘adolescent’ to refer to biological markers of age, but as culturally defined and unstable categories. See Ludmilla Jordanova, ‘Children in History: Concepts of Nature and Society’, in Geoffrey Scarre (ed.), *Children, Parents and Politics* (Cambridge: Cambridge University Press, 1989), pp. 3–24.

5 A point also made by Jana Funke, ‘“We Cannot Be Greek Now”: Age Difference, Corruption of Youth and the Making of *Sexual Inversion*’, *English Studies* 94 (2013), pp. 139–53; Diederik F. Janssen, ‘“Chronophilia”: Entries of Erotic Age Preference into Descriptive Psychopathology’, *Medical History* 59 (2015), pp. 575–98; Janssen, ‘Uranismus’. On age as an important analytical category in the history of sexuality, see also Lynn A. Botelho, ‘Age and History as Categories for Analysis: Refiguring Old Age’, *Age Culture Humanities* 1 (2014), unpaginated. [<https://ageculturehumanities.org/WP/age-and-history-as-categories-of-analysis-refiguring-old-age/>].

6 See e.g. Timothy D'Arch Smith, *Love in Earnest: Some Notes on the Lives and Writings of English ‘Uranian’ Poets from 1889 to 1930* (London: Routledge, 1970); Robert Aldrich, *The Seduction of the Mediterranean: Writing, Art and Homosexual Fantasy* (London: Routledge, 1993); Julie Anne Taddeo, ‘Plato's Apostles: Edwardian Cambridge and the “New Style of Love”’, *Journal of the History of Sexuality* 8 (1997), pp. 196–228; M. M. Kaylor, *Secreted Desires: The Major Uranians: Hopkins, Pater and Wilde* (Brno: Masaryk University, 2006); Matt Cook, *London and the Culture of Homosexuality, 1885–1914* (Cambridge: Cambridge University Press, 2008), pp. 122–42; Robert Deam Tobin, *Peripheral Desires: The German Discovery of Sex* (Philadelphia: University of Pennsylvania Press, 2015), pp. 53–82.

7 See e.g. Linda Dowling, *Hellenism and Homosexuality in Victorian Oxford* (Ithaca: Cornell University Press, 1994); Daniel Orrells, *Classical Culture and Modern Masculinity* (Oxford: Oxford University Press, 2011); Stefania Arcara, ‘Hellenic Transgressions, Homosexual Politics: Wilde, Symonds and Sicily’, *Studies in Travel Writing* 16 (2012), pp. 135–47; D. H. Mader and Gert Hekma, ‘Same Sex, Different Ages: On Pederasty in Gay History’, in Thomas K. Hubbard and Beert C. Verstraete (eds), *Censoring Sex Research: The Debate over Male Intergenerational Relations* (Walnut Creek: Left Coast Press, 2013), pp. 161–92.

8 See e.g. Sheila Rowbotham, *Edward Carpenter: A Life of Liberty and Love* (London: Verso, 2009), p. 199; 208–9; Sebastian Matzner, ‘From Uranians to Homosexuals: Philhellenism, Greek Homoeroticism and Gay Emancipation in Germany 1835–1915’, *Classical Receptions Journal* 2 (2010), pp. 60–91, here p. 68.

9 On damaging attacks linking homosexuals to child abuse, see e.g. Walter Fähnders, ‘Anarchism and Homosexuality in Wilhelmine Germany: Senna Hoy, Erich Mühsam, John Henry Mackay’, *Journal of Homosexuality* 29 (1995), pp. 117–54, here p. 130. On the ways in which such pejorative views continued to shape understandings of homosexuality throughout the twentieth century, see Jennifer Terry, *An American Obsession: Science, Medicine, and Homosexuality in Modern Society* (Chicago: University of Chicago Press, 1999), pp. 315–28.

10 Age is not considered in influential accounts of the emergence of modern homosexuality, e.g. Michel Foucault, *The History of Sexuality: The Will to Knowledge*, tr. Robert Hurley (London: Penguin, 1998); Arnold I. Davidson, *The Emergence of Sexuality: Historical Epistemology and the Formation of Concepts* (Cambridge: Harvard University Press, 2001).

11 On sexual science and childhood, see e.g. Stephen Kern, ‘Freud and the Discovery of Child Sexuality’, *History of Childhood Quarterly* 1 (1973), pp. 117–41; Lutz Sauerteig, ‘Loss of Innocence: Albert Moll, Sigmund Freud and the Invention of Childhood Sexuality around 1900’, *Medical History* 56 (2012), pp. 156–83; Heike Bauer, *The Hirschfeld Archives: Violence, Death, and Modern Queer Culture* (Philadelphia: Temple University Press, 2017), pp. 57–77; Katie Sutton, *Sex Between Body and Mind: Encounters Between Psychoanalysis and Sexual Science in the German‐Speaking World and Beyond, 1890s–1930s* (Ann Arbor: University of Michigan Press, forthcoming), chapter 1.

12 On the sciences of the child, see e.g. Sally Shuttleworth, *The Mind of the Child: Child Development in Literature, Science, and Medicine, 1840–1900* (Oxford: Oxford University Press, 2010); Alysa Levene, ‘Childhood and Adolescence’, in Mark Jackson (ed.), *The Oxford Handbook of the History of Medicine* (Oxford: Oxford University Press, 2011), pp. 321–37.

13 Carol Smart, ‘A History of Ambivalence and Conflict in the Discursive Construction of the “Child Victim” of Sexual Abuse’, *Social & Legal Studies* 8 (1999), pp. 391–409; Louise A. Jackson, *Child Sexual Abuse in Victorian England* (London: Routledge, 2001).

14 On mis‐readings of sexual assault cases as proto‐homosexuality, see Stephen Robertson, ‘Shifting the Scene of the Crime: Sodomy and the American History of Sexual Violence’, *Journal of the History of Sexuality* 19 (2010), pp. 223–42. On male victims of sexual assault (and an argument that this concept did not exist in the US until the twentieth century), see Stephen Robertson, ‘“Boys, of Course, Cannot be Raped”: Age, Homosexuality and the Redefinition of Sexual Violence in New York City, 1880–1955’, *Gender & History* 18 (2006), pp. 357–79.

15 For a bibliography, see Mirja Satka and Caroline Skehill, ‘History of Child Welfare and Child Protection in Europe’, in E. J. Mullen (ed.), *Oxford Bibliographies in Social Work* (Oxford: Oxford University Press, 2011) [<http://www.oxfordbibliographies.com/view/document/obo-9780195389678/obo-9780195389678-0052.xml>].

16 R. Danielle Egan and Gail L. Hawkes, ‘Imperiled and Perilous: Exploring the History of Childhood Sexuality’, *Journal of Historical Sociology* 21 (2008), pp. 355–67; Victoria Bates, *Sexual Forensics in Victorian and Edwardian England: Age, Crime and Consent in the Courts* (Basingstoke: Palgrave, 2016). On working‐class boys, see Seth Koven, ‘“From Rough Lads to Hooligans”: Boy Life, National Culture and Social Reform’, in Mary Russo, Andrew Parker, Doris Sommer, and Patricia Yaeger (eds), *Nationalisms and Sexualities* (New York, London: Routledge, 1992), pp. 365–91.

17 Nayan Shah, *Stranger Intimacy: Contesting Race, Sexuality and the Law in the North American West* (Berkeley: University of California Press, 2011), p. 132, characterises this as a ‘cataclysmic shift in social consciousness and legal protections for girls and their relations with adult men’. On the age of consent, see e.g. Mrinalini Sinha, *Colonial Masculinity: The ‘Manly Englishman’ and the ‘Effeminate Bengali’ in the Late Nineteenth Century* (Manchester: Manchester University Press, 1995), pp. 138–72; Helmut Graupner, ‘Sexual Consent: the Criminal Law in Europe and Overseas’, *Archives of Sexual Behavior* 29 (2000), pp. 415–61.

18 See e.g. Stephen Robertson, *Crimes Against Children: Sexual Violence and Legal Culture in New York City, 1880–1960* (Chapel Hill: University of North Carolina Press, 2005); Ishita Pande, ‘Sorting Boys and Men: Unlawful Intercourse, Boy‐Protection and the Child Marriage Restraint Act in Colonial India’, *Journal of the History of Childhood and Youth* 6 (2013), pp. 332–58.

19 Don Romesburg, ‘Making Adolescence More or Less Modern’, in Paula S. Fass (ed.), *The Routledge History of Childhood in the Western World* (London and New York: Routledge, 2013), pp. 229–38, here p. 235.

20 Angus McLaren, *The Trials of Masculinity: Policing Sexual Boundaries, 1870–1930* (Chicago: University of Chicago Press, 1997), p. 30; Jackson, *Child Sexual Abuse*, p. 105.

21 Tardieu's work was widely cited by e.g. Richard von Krafft‐Ebing, Caesare Lombroso, Sigmund Freud and Albert Moll, all of whom discussed sexual assaults on boys as well as girls. See Allen Esterson, ‘Jeffrey Masson and Freud's Seduction Theory: A New Fable Based on Old Myths’, *History of the Human Sciences* 11 (1998), pp. 1–21, here p. 12.

22 On the assumption that pederasty involved male youths, see Diederik F. Janssen, ‘Karl Heinrich Ulrichs: First Theorist of Erotic Age Orientation’, *Journal of Homosexuality* 64 (2017), pp. 1850–71, here p. 1851.

23 Ludwig Julius Caspar Mende, *Ausführliches Handbuch der Gerichtlichen Medizin* (Leipzig: Dut'sche Buchhandlung, 1826), p. 506. See also Ross Brooks, ‘“Vices Once Adopted”: Theorising Male Homoeroticism in German‐Language Legal and Forensic Discourses, 1752–1869’, *Reinvention: An International Journal of Undergraduate Research* 1 (2008). [<https://warwick.ac.uk/fac/cross_fac/iatl/reinvention/issues/volume1issue2/brooks/>].

24 Arthur Schopenhauer, *Die Welt als Wille und Vorstellung, vol* 2 (Leipzig: F. A. Brockhaus, 1888), pp. 643–51, here pp. 648–9.

25 Schopenhauer, *Die Welt*, p. 648. See also Bryan Magee, *The Philosophy of Schopenhauer* (Oxford: Oxford University Press, 1997), pp. 346–9. The emergence of the ‘sexually depraved’ older person in sexual science is traced by Kazuki Yamada, PhD Dissertation (University of Exeter, forthcoming).

26 Richard Francis Burton, *Terminal Essay: D ‐ Pederasty, A Plain and Literal Translation of the Arabian Nights’ Entertainments, Now Entitled the Book of the Thousand Nights and a Night; with Introduction, Explanatory Notes on the Manners and Customs of Moslem Men, and a Terminal Essay Upon the History of the Nights, Vol. X* (London: Burton Club for Private Subscribers Only, 1886), pp. 205–54, here e.g. pp. 205–6, 236–8, 241–2.

27 A French Army‐Surgeon [Dr Jacobus X], *Untrodden Fields of Anthropology: Observations on the Esoteric Manners and Customs of Semi‐Civilized Peoples* (New York: Privately Issued by the American Anthropological Society [Charles Carrington], undated), here pp. 91–104, 266–7. An expanded 1896 translation of a French text entitled *Amour aux Colonies* (1893), it included copious references to works of sexual science. See Sarah Bull, ‘A Purveyor of Garbage? Charles Carrington and the Marketing of Sexual Science in Late‐Victorian Britain’, *Victorian Review* 38 (2012), pp. 55–76. Similar representations are found across orientalist and colonial writing. See Rudi Bleys, *The Geography of Perversion: Male‐to‐Male Sexual Behaviour Outside the West and the Ethnographic Imagination, 1750–1918* (London: Cassell, 1996).

28 Sexual scientists were troubled by this linguistic ambiguity, see e.g. Albert Moll, *Die conträre Sexualempfindung* (Berlin: H. Kornfeld, 1891), pp. 5–6; Hirschfeld, *Die Homosexualität*, p. 12.

29 See Foucault, *History of Sexuality*, pp. 104–5; Beth Bailey, ‘The Vexed History of Children and Sex’, in Paula S. Fass (ed.), *The Routledge History of Childhood in the Western World* (Abingdon, New York: Routledge, 2013), pp. 191–210.

30 Jacobus X, *Untrodden Fields*, p. 96.

31 Sauerteig, ‘Loss of Innocence’, pp. 156–83.

32 See e.g. John Demos and Virginia Demos, ‘Adolescence in Historical Perspective’, *Journal of Marriage and Family* 31 (1969), pp. 632–8; Alice Boardman Smuts, *Science in the Service of Children: 1893–1935* (New Haven: Yale University Press, 2006).

33 Iwan Bloch, *Das Sexualleben unserer Zeit in seinen Beziehungen zur modernen Kultur* (Berlin: Louis Marcus, 1907), p. 516.

34 On Hellenism and sexology, see Funke, ‘Age Difference’; Matzner, ‘Uranians to Homosexuals’; S. Kool, ‘A Matrix of Interests: Freud, the Sexologists, and the Legacy of Greece’, *Akroterion* 58 (2013), pp. 79–96.

35 On Wilde as corrupter of youth, see e.g. Ari Adut, ‘A Theory of Scandal: Victorians, Homosexuality, and the Fall of Oscar Wilde’, *American Journal of Sociology* 111 (2005), pp. 213–48. See Orrells, *Classical Culture*, pp. 185–218, on the (misguided) Hellenism of Wilde's defence.

36 John Marshall, ‘Pansies, Perverts and Macho Men: Changing Conceptions of Male Homosexuality’, in Kenneth Plummer (ed.), *The Making of the Modern Homosexual* (London: Hutchinson, 1981), pp. 133–54, here p. 141.

37 See e.g. James D. Steakley, ‘Iconography of a Scandal: Political Cartoons and the Eulenburg Affair in Wilhelmine Germany’, in Martin B. Duberman, Martha Vicinus and George Chauncey (eds), *Hidden from History: Reclaiming the Gay and Lesbian Past* (London: Penguin, 1991), pp. 233–63; Harry G. Cocks, *Nameless Offences: Homosexual Desire in the 19th Century* (London: I.B. Tauris, 2010); Norman Domeier, *The Eulenburg Affair: A Cultural History of Politics in the German Empire* (Rochester: Camden House, 2015).

38 On Symonds and age, see Stefano Maria Evangelista, ‘Platonic Dons, Adolescent Bodies: Benjamin Jowett, John Addington Symonds, Walter Pater’, in George Rousseau (ed.), *Children and Sexuality: From the Greeks to the Great War* (Basingstoke: Palgrave, 2007), pp. 206–36, here pp. 218–25; Orrells, *Classical Culture*, pp. 146–84; Funke, ‘Age Difference’. On Symonds' memoirs, see Amber K. Regis, ‘Introduction’, in Amber K. Regis (ed.), *The Memoirs of John Addington Symonds: A Critical Edition* (Basingstoke: Palgrave, 2016), pp. 1–56.

39 Symonds’ friend Edward Carpenter (and others) offered a different response to this problem, as shown below.

40 Funke, ‘Age Difference’.

41 Marc‐André Raffalovich, *Uranisme et unisexualité* (Lyon and Paris: Storck, 1896), p. 241. See also P. W. J. Healy, ‘*Uranisme et unisexualité*: A Late Victorian View of Homosexuality’, *New Blackfriars* 59 (1978), pp. 56–65, here p. 59.

42 E.g. ‘konträres Geschlechtsgefühl’ [contrary sexual feeling]; ‘sexual inversion’; ‘unisexualité’ [unisexualism]; ‘homogenic love’; ‘gleichgeschlechtliche Liebe’ [same‐sex love]; ‘Parisexualität’ [adult male homosexuality]. See also Ralph Matthew Leck, *Vita Sexualis: Karl Ulrichs and the Origins of Sexual Science* (Chicago: University of Illinois Press, 2016), pp. 62–3.

43 Karl Heinrich Ulrichs, *Forschungen über das Räthsel der mannmännlichen Liebe* (Leipzig: Heinrich Matthes, 1864), pp. 1–2.

44 Leck, *Vita Sexualis*, pp. 38–9; Tobin, *Peripheral Desires*, p. 11.

45 Johann Ludwig Casper, *Practisches Handbuch der gerichtlichen Medizin* (Berlin: August Hirschwald, 1860), p. 182.

46 Albert Moll, *Die Conträre Sexualempfindung* (Berlin: Fischer’ Medicin Buchhandlung, 1899), pp. 101–2.

47 Edward Carpenter's translation of Moll, *Conträre Sexualempfindung*, p. 101, ‘Research Notebooks’, Sheffield Archives, C.C. MSS 93. Carpenter, ‘Research Notebook 1912’, Sheffield Archives, C.C. MSS 284, pp. 21‐3.

48 Hirschfeld, *Homosexualität*, p. 7.

49 Hirschfeld, *Homosexualität*, pp. 977–91. See also Mancini, *Sexual Freedom*, pp. 72, 154.

50 Havelock Ellis, *Sexual Inversion: A Critical Edition* (Basingstoke: Palgrave, 2008), p. 218.

51 Hirschfeld, *Homosexualität*, p. 989.

52 Magnus Hirschfeld, ‘Vom Wesen der Liebe: Zugleich ein Beitrag zur Lösung der Frage der Bisexualität’, *Jahrbuch für sexuelle Zwischenstufen* 8 (1906), pp. 1–284, here p. 284; Bloch, *Sexualleben*, p. 726.

53 Ulrichs, *Forschungen über das Räthsel*, pp. 1–2.

54 Gustav Jaeger, *Die Entdeckung der Seele* (Leipzig: Ernst Günther, 1880), p. 264.

55 Bloch, *Sexualleben*, p. 544.

56 Hirschfeld, *Homosexualität*, p. 993.

57 John Addington Symonds, *A Problem in Modern Ethics: Being an Enquiry into the Phenomenon of Sexual Inversion, Addressed Especially to Medical Psychologists and Jurists* (London: Privately Published, 1896), p. 103.

58 Symonds, *Modern Ethics*, p. 15; Bloch, *Sexualleben*, p. 544.

59 Richard von Krafft‐Ebing, ‘Ueber Unzucht mit Kindern und Pädophilia Erotica’, *Friedreichs Blätter für gerichtlichte Medizin und Sanitätzpolizei* 47 (1896), pp. 261–83. The term appeared in the tenth German edition of *Psychopathia Sexualis*. See also, Ambroise Tardieu, *Étude médico‐légale sur les attentats aux moeurs* (Paris: J. B. Baillière, 1873), pp. 209–10.

60 Albert Moll, *Das Sexualleben des Kindes* (Leipzig: F. C. W. Vogel, 1908), p. 199.

61 When Hirschfeld reported on the 1862 case of Johann Baptist von Schweizer, who had been arrested in a park in Mannheim with a companion who was probably a fourteen‐year‐old boy, he did not specify the boy's age. See Hubert Kennedy, ‘Johann Baptist Von Schweitzer’, *Journal of Homosexuality* 29 (1995), pp. 69–96, here, p. 86.

62 Karl Heinrich Ulrichs, *Incubus: Urningsliebe und Blutgier* (Leipzig: A. Serbe, 1869), p. 28.

63 Hirschfeld, *Homosexualität*, p. 281. Hirschfeld also introduced the concept of ‘infantilism’ in the 1910s to argue that sexual attraction to children was experienced only by a small minority of homosexual (and heterosexual) individuals. See e.g. Hirschfeld, *Homosexualität*, pp. 302–4.

64 E.g. Steven Angelides, ‘The Emergence of the Paedophile in the Late Twentieth Century’, *Australian Historical Studies* 137 (2005), pp. 272–95; Matthew Thomson, *Lost Freedom: The Landscape of the Child and the British Post‐War Settlement* (Oxford: Oxford University Press, 2013), pp. 153–83.

65 On sexology and aetiology, see Valerie Rohy, *Lost Causes: Narrative, Etiology, and Queer Theory* (Oxford: Oxford University Press, 2015); Benjamin Kahan, *The Book of Minor Perverts: Sexology, Etiology, and the Emergences of Sexuality* (Chicago: Chicago University Press, 2019).

66 See Kate Fisher and Jana Funke, ‘Cross‐Disciplinary Translations: British Sexual Science, History and Anthropology’, in Heike Bauer (ed.), *Sexology and Translation: Cultural and Scientific Encounters Across the Modern World* (Philadelphia: Temple University Press, 2015), pp. 95–114; Kate Fisher and Jana Funke, ‘“Let Us Leave the Hospital; Let Us Go On a Journey Around the World”: British and German Sexual Science and the Global Search for Sexual Variation’, in Veronica Füchtner, Douglas E. Haynes and Ryan M. Jones (eds), *A Global History of Sexual Science: 1880–1960* (Berkeley: University of California Press, 2018), pp. 51–69. On sexology and anthropology, see also Andrew P. Lyons and Harriet D. Lyons, *Irregular Connections: A History of Anthropology and Sexuality* (Lincoln: University of Nebraska Press, 2004), pp. 155–84.

67 Sean Brady (ed.), *John Addington Symonds (1840–1893) and Homosexuality: A Critical Edition of Sources* (Basingstoke: Palgrave Macmillan, 2012), p. 223.

68 Edvard Westermarck, *The Origin and Development of the Moral Ideas*, vol. 2 (London: Macmillan, 1917), p. 468. On Westermarck and homosexuality, see e.g. Juhani Ihanus, *Multiple Origins: Edward Westermarck in Search of Mankind* (Frankfurt am Main: Peter Lang, 1999), pp. 226–9; Ralph Leck, ‘Westermarck's Morocco’, in Füchtner, et al. (eds), *A Global History of Sexual Science*, pp. 70–96.

69 Iwan Bloch, *Beiträge zur Aetiologie der Psychopathia Sexualis* (Dresden: H. R. Dohrn, 1902), p. 32.

70 E.g. Friedrich Krauss, ‘Von den Uraniern’, *Anthropophyteia* 6 (1909), pp. 177–80, used anthropological evidence to support homosexual reform. See also Leck, *Vita Sexualis*, p. 62.

71 Havelock Ellis quoted in Erwin J. Haeberle, *75 Years of Sexology (1908–1983)* (Washington: World Association for Sexology, 1983), p. 15.

72 See e.g. Ellis, *Sexual Inversion*, p. 104. See also, Dan Orrells, ‘Greek Love, Orientalism and Race: Intersections in Classical Reception’, *The Cambridge Classical Journal* 58 (2012), pp. 194–220.

73 Bloch, *Sexualleben*, p. 541; Hirschfeld, *Homosexualität*, pp. 296–7.

74 Ellis, *Sexual Inversion*, p. 128. See also, e.g. Hirschfeld, *Homosexualität*, pp. 42–6, p. 110.

75 Symonds, *Modern Ethics*, p. 94.

76 E.g. cases VI, IX, XII in Ellis, *Sexual Inversion*, pp. 132, 136, 138.

77 Symonds, *Modern Ethics*, p. 93.

78 On Freud's views on homosexuality, see e.g. Sara Flanders et al., ‘On the Subject of Homosexuality: What Freud Said’, *The International Journal of Psychoanalysis* 97 (2016), pp. 933–50. On Freud and childhood sexuality, see Kern, ‘Child Sexuality’; Sauerteig, ‘Loss of Innocence’.

79 In his 1910 case study of Leonardo da Vinci, Freud suggested that da Vinci's homosexuality was caused by him being ‘kissed by her [the mother] into precocious sexual maturity’ (Sigmund Freud, ‘Leonardo da Vinci and a Memory of his Childhood’ in Sigmund Freud, *The Standard Edition of the Complete Psychological Works of Sigmund Freud*, vol. XI, (1957: London: Vintage, 2001), pp. 59–137, here p. 131). On Freud and developmental arrest, see Flanders et al., ‘Homosexuality’, pp. 944–5.

80 Scholars frequently argue that the question of whether homosexuality was inborn or acquired divided sexology and psychoanalysis, e.g. Ivan Crozier, ‘Taking Prisoners: Havelock Ellis, Sigmund Freud and the Construction of Homosexuality, 1897–1951’, *Social History of Medicine* 13 (2000), pp. 447–66; Mancini, *Hirschfeld*, pp. 71–7. While not inaccurate, sexologists were open to exploring the impact of external influences in causing homosexuality, and Freud did not rule out congenital factors. See Timothy F. Murphy, ‘Freud and Sexual Reorientation Therapy’, *Journal of Homosexuality* 23 (1992), pp. 21–38, here p. 24. Most importantly, a shared interest in childhood sexual development connected sexology and psychoanalysis. On this point, see also Sauerteig, ‘Loss of Innocence’; Sutton, *Between Body and Mind*, chapter 1.

81 E.g. Hirschfeld, *Homosexualität*, pp. 46–7; 344–5.

82 Hirschfeld, *Homosexualität*, p. 46–8, 46.

83 Hirschfeld, *Homosexualität*, pp. 46–8.

84 E.g. Albert Moll, *Die Conträre Sexualempfindung* (Berlin: H. Kornfeld, 1891), pp. 165–6. In Moll, *Das Sexualleben des Kindes*, p. 206, he explained further ‘that the child's sexual interest can be turned towards the same‐sex during this period of undifferentiated sexuality, so that a permanent perversion might develop’, especially when young people enter into relationships with adults. Moll initially supported Hirschfeld in the fight to decriminalise male homosexuality, but later declared that he had underestimated the problem of young people being seduced into homosexuality. See Volkmar Sigusch, ‘The Sexologist Albert Moll – Between Sigmund Freud and Magnus Hirschfeld’, *Medical History* 56 (2012), pp. 184–200, here pp. 195–6.

85 Moll, *Conträre Sexualempfindung*, pp. 127–8.

86 Arthur Kronfeld, *Über psychosexuellen Infantilismus: eine Konstitutionsanomalie* (Leipzig: Ernst Bircher, 1921), p. 20.

87 See e.g. George Ives, ‘Diaries’, vols. 31‐5, 9 July 1899, Harry Ransom Center, University of Texas at Austin, George Cecil Ives Papers, p. 49 (3908), in response to reading Carpenter. This view is expressed regularly in other diary entries.

88 Some homophile writers adopted a ‘masculinist’ model to oppose conceptualisations of the effeminate inborn homosexual who is exclusively attracted to men. See e.g. Tobin, *Peripheral Desires*, pp. 53–82.

89 On *Die Gemeinschaft*, see e.g. Harry Oosterhuis and Hubert Kennedy (eds), *Homosexuality and Male Bonding in Pre‐Nazi Germany: The Youth Movement, the Gay Movement, and Male Bonding before Hitler's Rise: Original Transcripts from ‘Der Eigene’, the First Gay Journal in the World* (New York: Haworth Press, 1991); Yvonne Ivory, ‘The Urning and His Own: Individualism and the Fin‐de‐Siècle Invert’, *German Studies Review* 26 (2003), 333–52; Claudia Bruns, *Politik des Eros: der Männerbund in Wissenschaft, Politik und Jugendkultur, 1880–1934* (Cologne: Böhlau, 2008).

90 John Henry Mackay, *Fenny Skaller and Other Prose Writings from the Books of the Nameless Love* (Amsterdam: Southernwood, 1988), pp. 160, 178. On Mackay, see Hubert C. Kennedy, *Anarchist of Love: The Secret Life of John Henry Mackay* (New York: Mackey Society, 1983); Fähnders, ‘Anarchism and Homosexuality’; J. Edgar Bauer, ‘On the Nameless Love and Infinite Sexualities: John Henry Mackay, Magnus Hirschfeld and the Origins of the Sexual Emancipation Movement’, *Journal of Homosexuality* 50 (2005) pp. 1–26. For other (often ignored) writers who were explicit champions of age‐discrepant sexual relationships, see Mader and Hekma, ‘Same Sex, Different Ages’.

91 On the alleged antagonism between homophile movements and sexology, see e.g. Oosterhuis and Kennedy (eds), *Homosexuality and Male Bonding*; Dowling*, Hellenism*; Ivory, ‘The Urning’; Tobin, *Peripheral Desires*.

92 E.g. Benedict Friedlaender, *Denkschrift verfasst für die Freunde und Fondszeichner des Wissenschaftlich‐Humanitären Komitees* (Berlin: Printed Manuscript, 1907), p. 32; Benedict Friedländer, *Renaissance des Eros Uranios: die physiologische Freundschaft, ein normaler Grundtrieb des Menschen und eine Frage der männlichen Gesellungsfreiheit* (Berlin: Otto Lehmann, 1904), p. 191–3.

93 When the second statistical survey of 1904 failed to identify significant numbers of bisexuals, Friedländer cautiously embraced the inborn model. See Friedländer, *Eros Uranios: Addendum*, pp. 72–3.

94 Friedländer, *Eros Uranios*, pp. 211–54. For more on Friedländer's work on friendship, see Marita Keilson‐Lauritz, ‘Benedict Friedländer’, in Volkmar Sigusch and Günter Grau (eds), *Personenlexikon der Sexualforschung* (Frankfurt am Main: Campus, 2009), pp. 198–203.

95 Friedländer, *Eros Uranios*, 6. See also Benedict Friedländer, ‘Die physiologische Freundschaft als normaler Grundtrieb des Menschen und als Grundlage der Sozialität’, *Jahrbuch für sexuelle Zwischenstufen* 6 (1904), pp. 179–213.

96 Friedländer, *Eros Uranios*: *Addendum*, pp. 63–7; 72.

97 On this article, see Josephine Crawley Quinn and Christopher Brooke, ‘“Affection in Education”: Edward Carpenter, John Addington Symonds and the Politics of Greek Love’, *Oxford Review of Education* 37 (2011), pp. 683–98; Rowbotham, *Carpenter*, p. 200. The article was reprinted in Carpenter's influential *The Intermediate Sex*; Edward Carpenter, *The Intermediate Sex* (London: Swan Sonnenschein & Co., 1908). Edward Carpenter, ‘Affection in Education’, *International Journal of Ethics* 9 (1899), pp. 482–94, here p. 484.

98 Bloch, *Sexualleben*, pp. 693–4, p. 725.

99 Moll, *Sexualleben*, p. 177.

100 Writers also stressed the benefit of age‐differentiated bonds for the older partner, e.g. Carpenter ‘Affection in Education’, p. 484; Friedländer, *Eros Uranios*, p. 156.

101 British Sexological Society, ‘Letters Received’: Adolf Brand, 29 November 1933, Harry Ransom Center, University of Texas at Austin, British Sexological Society Records (Box 4.2, MS 00518), p. 3. See also e.g. Elisarion von Kupffer, ‘The Ethical‐Political Significance of *Lieblingsminne*’, *Journal of Homosexuality* 22 (1892), pp. 35–48, here pp. 36–7 (originally published in *Der Eigene* in 1899); Friedländer, *Eros Uranios*, pp. 74–83.

102 Carpenter, ‘Affection in Education’, p. 485.

103 Edward Carpenter, *Intermediate Types Among Primitive Folk: A Study in Social Evolution* (London: George Allen & Unwin, 1919), pp. 134–5.

104 See e.g. Friedländer, *Eros Uranios*, p. 156; von Kupffer, ‘Ethical‐Political Significance’, 41–2. On the *Free School Movement*, see e.g. Thijs Maasen, ‘Man‐Boy Friendships on Trial’, *Journal of Homosexuality* 20 (1991), pp. 47–70; John Alexander Williams, ‘Ecstasies of the Young: Sexuality, the Youth Movement, and Moral Panic in Germany on the Eve of the First World War’, *Central European History* 34 (2001), pp. 163–89.

105 This argument was central to e.g. Friedländer, ‘Physiologische Freundschaft’; von Kupffer, ‘Ethical‐Political Significance’; Carpenter, ‘Affection in Education’. See also Harry Oosterhuis, ‘Homosexual Emancipation in Germany Before 1933’, *Journal of Homosexuality* 22 (1992), pp. 1–28, here p. 16; Crawley and Quinn, ‘Affection in Education’, pp. 690, 693.

106 Friedländer, ‘Physiologische Freundschaft’, pp. 182–3, hoped his article might reconcile Hirschfeld's medical approach and the literary‐historical stance of von Kupffer and others.

107 In a preface to the article, Hirschfeld acknowledged the relevance of Friedländer's views to the scientific study of homosexuality; see Friedländer, ‘Physiologische Freundschaft’, p. 181.

108 Magnus Hirschfeld, ‘Vorbemerkung des Herausgebers’, *Jahrbuch für Sexuelle Zwischenstufen* 6 (1904), p. 181.

109 Numa Praetorius [Eugène Wilhelm], ‘Die Bibliographie der Homosexualität für das Jahr 1899’, *Jahrbuch für Sexuelle Zwischenstufen* 2 (1900), p. 387.

